# The effect of spatial aggregation on performance when mapping a risk of disease

**DOI:** 10.1186/1476-072X-13-9

**Published:** 2014-03-13

**Authors:** Caroline Jeffery, Al Ozonoff, Marcello Pagano

**Affiliations:** 1 Liverpool School of Tropical Medicine, Department of International Public Health, Monitoring and Evaluation Technical assistance and Research group, Liverpool L3 5QA, UK; 2 Department of Pediatrics, Harvard Medical School, Center for Patient Safety and Quality Research, Boston Children’s Hospital, Boston, MA 02118, USA; 3 Department of Biostatistics, Harvard School of Public Health, Boston, MA 02115, USA

**Keywords:** Disease risk mapping, Distance-based mapping, Spatial data, Aggregation effect, Scale effect, MAUP, Simulations, Spatial epidemiology

## Abstract

**Background:**

Spatial data on cases are available either in point form (e.g. longitude/latitude), or aggregated by an administrative region (e.g. zip code or census tract). Statistical methods for spatial data may accommodate either form of data, however the spatial aggregation can affect their performance. Previous work has studied the effect of spatial aggregation on cluster detection methods. Here we consider geographic health data at different levels of spatial resolution, to study the effect of spatial aggregation on disease mapping performance in locating subregions of increased disease risk.

**Methods:**

We implemented a non-parametric disease distance-based mapping (DBM) method to produce a smooth map from spatially aggregated childhood leukaemia data. We then simulated spatial data under controlled conditions to study the effect of spatial aggregation on its performance. We used an evaluation method based on ROC curves to compare performance of DBM across different geographic scales.

**Results:**

Application of DBM to the leukaemia data illustrates the method as a useful visualization tool. Spatial aggregation produced expected degradation of disease mapping performance. Characteristics of this degradation, however, varied depending on the interaction between the geographic extent of the higher risk area and the level of aggregation. For example, higher risk areas dispersed across several units did not suffer as greatly from aggregation. The choice of centroids also had an impact on the resulting mapping.

**Conclusions:**

DBM can be implemented for continuous and discrete spatial data, but the resulting mapping can lose accuracy in the second setting. Investigation of the simulations suggests a complex relationship between performance loss, geographic extent of spatial disturbances and centroid locations. Aggregation of spatial data destroys information and thus impedes efforts to monitor these data for spatial disturbances. The effect of spatial aggregation on cluster detection, disease mapping, and other useful methods in spatial epidemiology is complex and deserves further study.

## Background

As John Snow demonstrated so effectively with the cluster of cholera cases around the Broad Street pump, geographic location of disease cases can be a crucial first step to identify and eventually prevent the source of disease outbreaks [1,2]. We continue this tradition in the modern practice of public health, where understanding the epidemiology of disease informs the design of population-level interventions and policies. Spatial information on cases can reveal the spread of disease across a region, or it can be incorporated in statistical analyses [3-5], but these data are complex with many characteristics that might influence analysis [6]. In particular the level of precision of the spatial data is an aspect with immediate analytic considerations, and several studies have shown that accurately identifying the geographic spread of a spatial disturbance depends upon the level of precision of the spatial data [7-9].

When case locations are reported, they are available in either point or aggregated form. For the former, the location is given by a pair of coordinates (*x; y*), e.g. referring to Cartesian coordinates or to longitude and latitude. We refer to them as *continuous* spatial data. Alternatively, the location of a case can be given in aggregated form as a subset of the study region called the *aggregation unit*, typically an administrative level such as ZIP code or census tract. The subregion is often available with a centroid represented by a pair of coordinates. The centroid describes the ‘center’ of the unit, and is typically chosen to be the location of the administrative or geographic center of the unit. This coarser spatial data on cases can result from the collection process or might have been intentionally aggregated due to privacy. We call this form of data *discrete* spatial data. Measuring the *effect of spatial aggregation* is the study of determining how the performance of a statistical method is affected when the spatial data are available in a discrete fashion rather than continuous (e.g. ZIP code vs longitude and latitude), or in one discrete level versus another (e.g. ZIP code vs census tract) [10]. This effect is also termed *effect of scale* or *effect of discretisation*, and is a particular aspect of the Modifiable Areal Unit Problem [10].

Spatial methods are usually classified into addressing one of three problems: cluster detection, clustering, and spatial variation in risk [11,12]. Several authors have studied the effect of spatial aggregation on some of the spatial methods used in disease surveillance (see [7] and references therein). Some studies have shown that cluster detection methods [12] applied to data aggregated at a coarser scale will result in a loss of statistical power. Most of these studies are limited in the number of spatial alternatives and the number of discrete aggregation levels considered. When the geographic or population size of the cluster varies, and when many levels of aggregation are considered, the impact on power can involve a complex relationship between the geographical extent of the cluster and that of the aggregation unit [7,9,13]. As an alternative to studying the relationship between an individual's location and the acquisition of disease, exploratory disease mapping methods estimate a change in the spatial distribution of cases from a reference distribution, which represents a residual spatial risk surface after all known risk factors have been accounted for [12,14]. With continuous spatial data, one can estimate the log ratio of two inhomogeneous Poisson process intensities [15,16], or a combined measure comparing the two distributions on several one-dimensional scales [17,18]. Both approaches define their estimator as a function mapping any point in the study region to a real value. With discrete spatial data, one usually determines a single value for each aggregated unit (e.g. rate, proportion), and statistical smoothing techniques are available to account for varying population sizes, influence of neighboring units, and other variables [12,14,19-23].

Regardless of the input data, the output of disease mapping is a set of values associated with a regular grid of points atop the study region. With discrete data, we typically assign a single estimate to all grid points in the same unit, or to the centroid of the unit only, using kriging or interpolation to define values at the remaining grid points [24]. The caveat is that this second approach may result in non-positive values [14]. Finally we use a color scale to visualize how the estimate varies across the study region.

In this paper we consider a non-parametric distance-based mapping (DBM) approach [17,18], and measure the effect of spatial aggregation on its performance. In the next section, we describe our methods in the context of continuous and discrete data. We then illustrate the utility of our approach with an application to childhood leukaemia data from upstate New York [25,26], and simulate data to compare estimation of a dichotomized risk in the unit disk under several levels of aggregation versus continuous data. We conclude with some observations and discussion on the advantages and limitations of our proposed approach.

## Methods

We consider spatial methods for the display or analysis of a quantitative variable across a study region. Typical examples might be a climate variable (e.g. temperature, rainfall); an environmental exposure (carbon monoxide concentration, PM10 pollution); or an epidemiologic measure (e.g. prevalence or cumulative incidence of a disease). The spatial data available might consist of a sampled measurement of the variable at several fixed locations, or captured locations of a particular event. In either case, we aim to estimate the variable of interest at any location in the study region, at which point we say such an estimate *maps* the variable across the study region. Here we focus on approaches that map a risk of disease, for which the available data consist either of locations or aggregated counts of diagnosed cases.

Several measures from classical epidemiology are commonly used to represent risk: incidence rate, rate ratio (RR), or odds ratio (OR) are well-known examples. Incidence is defined with respect to an underlying population and time period, while RR or OR compare rates or odds respectively between two distinct exposure groups. When defining a risk function on a study region, we frame the function and its estimation as a comparison of two components, *observed* and *expected*. For example, observed locations of cancer cases can be compared with a representative sample from the population-at-risk [25,27]. Likewise, ongoing spatial monitoring of syndromic surveillance data might compare locations of respiratory illness during a particular week to those reported previous weeks [13]. Heterogeneity of spatial distribution of cases reflects the underlying density of human population throughout the region, and this is incorporated appropriately into the expected component.

### Notation and definitions

Consider a study region *S*, a bounded subset of a two-dimensional Euclidean space, which simultaneously represents the spatial support for our data and the domain of the associated mapping function, and a finite set of grid points S’ which are chosen by superimposing a lattice over the study region:

Definition: Let *S’* = *{y*_1_,…,*y*_*r*_*}* in *S* be a finite collection of points in *R*^*2*^. We call any real-valued function *M*: *S’ → R* a *(disease risk) mapping function*, and we call the range of *M*, i.e. the set of function values {*M*(*y*_1_),…,*M*(*y*_*r*_)}, the set of *(disease) scores*.

In practice, we find it convenient to represent *S* by its finite subset *S’* , and will simply write *S* for the grid point subset wherever this is clear. The definition above extends in the natural way to regions *S* embedded within higher dimensional spaces, but we focus on applications which model spatial locations in the Euclidean plane.

Given a set of observations, an *estimator of M* is a real-valued function $\hat{M}$ defined on *S*. In our context, we define the sample space and the mapping function more precisely. When spatial data are *continuous*, we assume that each spatial location of a case is a single point in *S*. Conversely when spatial data are *discrete,* we suppose that *S* can be divided into *m* non-intersecting subregions {*S*_1_,…,*S*_*m*_} with each subregion labeled by a centroid point *z*_*i*_ in *S*_*i*_. The centroid refers to the geographic center of the unit. When the spatial data of each case is *discrete*, that case location is one of these subregions, along with its centroid, and we define the sample space as the collection of centroids {*z*_1_*,…,z*_*m*_}.

Finally we consider the bivariate random vector *X* drawn from an underlying probability distribution representing the location of cases in the continuous setting and let *f*: *R*^*2*^ *→ R* be the corresponding probability distribution function (PDF). We frame a *disease risk mapping function* as a comparison between *f* and a reference function *f*_*0*_: *R*^*2*^ *→ R* defined throughout the region *S*, where *f* and *f*_*0*_ represent respectively the ‘observed’ spatial distribution of cases and the ‘expected’ distribution based on population and other factors. In the discrete setting, we define similar PDF *f*^(*m)*^*:R*^*2*^ *→ R* and a disease risk mapping function is framed as a comparison of the observed *f*^(*m*)^ to an expected reference function $f_{0}^{\left(m\right)}$: *R*^*2*^ *→ R* throughout the region. We link the two settings by a function mapping locations to the corresponding subregion centroids. Thus we consider both discrete and spatial mapping within the same probabilistic framework which allows us to build upon previous work [17,18].

### Disease mapping for continuous and discrete data

We now suppose our data consist of disease case locations diagnosed during a specified time period. For each case we observe a location in *S*. The general motivation behind the mapping approach proposed by Jeffery et al. [17,18] is to avert the curse-of-dimensionality (CoD) by comparing the observed and expected spatial distributions in one dimension only. The CoD occurs if the performance of a statistical method is heavily diminished when applied to higher dimensional data, unless the sample size is increased beyond practical values. The phenomenon is explained by the fact that, when the dimension of the data increases, the volume of the sample space grows exponentially in comparison to the sample size [28,29]. While the CoD may be manageable in two dimensions, this approach generalizes naturally and without great penalty to higher dimensions, where the effect of the curse is much more severe. Any potential loss of information from the projection is recovered by considering multiple projections from different angles and combining these comparisons. Inspired by tomographic imaging, the two-dimensional space is studied as a fixed number of one-dimensional slices.

Briefly summarizing the methodology, we place a fixed number *N* of fixed points *c*_*i*_ along a circle circumscribed to *S*. Each point *c*_*i*_ defines a projection, where the one-dimensional counterpart of *X* is defined as the Euclidean distance *d = ||c*_*i*_*– X||*, with associated cumulative distribution function (CDF) *F*_*i*_(*d*) and *F*_0_*(d)*. The one-dimensional comparison of *F*_*i*_ and *F*_*0*_ is constructed as a function *γ*_*i*_: R *→* R estimated via numerical integration. The comparator *γ*_*i*_ is usually chosen as the difference between the two integrals but other functions, such as a weighted difference or a ratio, have been proposed. The widths of the projections are modified adaptively with a parameter *p*_*0*_ which acts as a smoothing value. The parameter p_0_ is common to all projections, and guarantees that they all play comparable roles prior to recombination. The final disease mapping function *Γ(y)* is defined as *Γ*: *R*^*2*^ *→* R for any *y* in *S* as the average of the one-dimensional projections *γ*_*i*_*(y)* around *y*. When observed equals expected, *Γ* is constant throughout the region and we say that the map is *flat*.

To define an estimator for *Γ*, we assume *F*_0_ remains known and suppose the locations of *n* cases are represented by random variables *X*_1_,…,*X*_*n*_, independently identically distributed according to *F*. For each projection *γ*_*i*_, we consider the one-dimensional empirical cumulative distribution function (ECDF) and use these to define consistent estimators for *γ*_*i*_(*y*) and *Γ(y)* for fixed *y* in *S*.

To adapt the framework above to discrete data, we replace the sample space *R*^*2*^ with the discrete collection of centroids {*z*_*1*_,…,*z*_*m*_}. Discrete analogues of the one-dimensional projections defined above are still valid and combine as before for a disease mapping function *Γ* with the typical desirable properties. For example some mild conditions ensure that the same properties of consistent and unbiased estimation guaranteed for the continuous mapping function will remain in place as well for discrete data. Application of the relative probability theory is straightforward, and mathematical derivations and necessary theorems are described elsewhere [17].

### Simulations

To illustrate our approach to discrete data, we first apply DBM to incident cases of leukaemia reported in the eight counties of the upper part of New York State between 1978-1982 [25,26]. We then evaluate DBM for discrete data with simulations in the unit disk. Performance is measured as the accuracy to locate an increased risk of disease when the underlying distribution is uniform. Data are first sampled from a continuous distribution, then discretized at four different levels. We study how accuracy changes across five datasets. All analysis was done using the software R [30], and the code is available upon request.

### Data and simulated outbreaks

**Figure 1:**
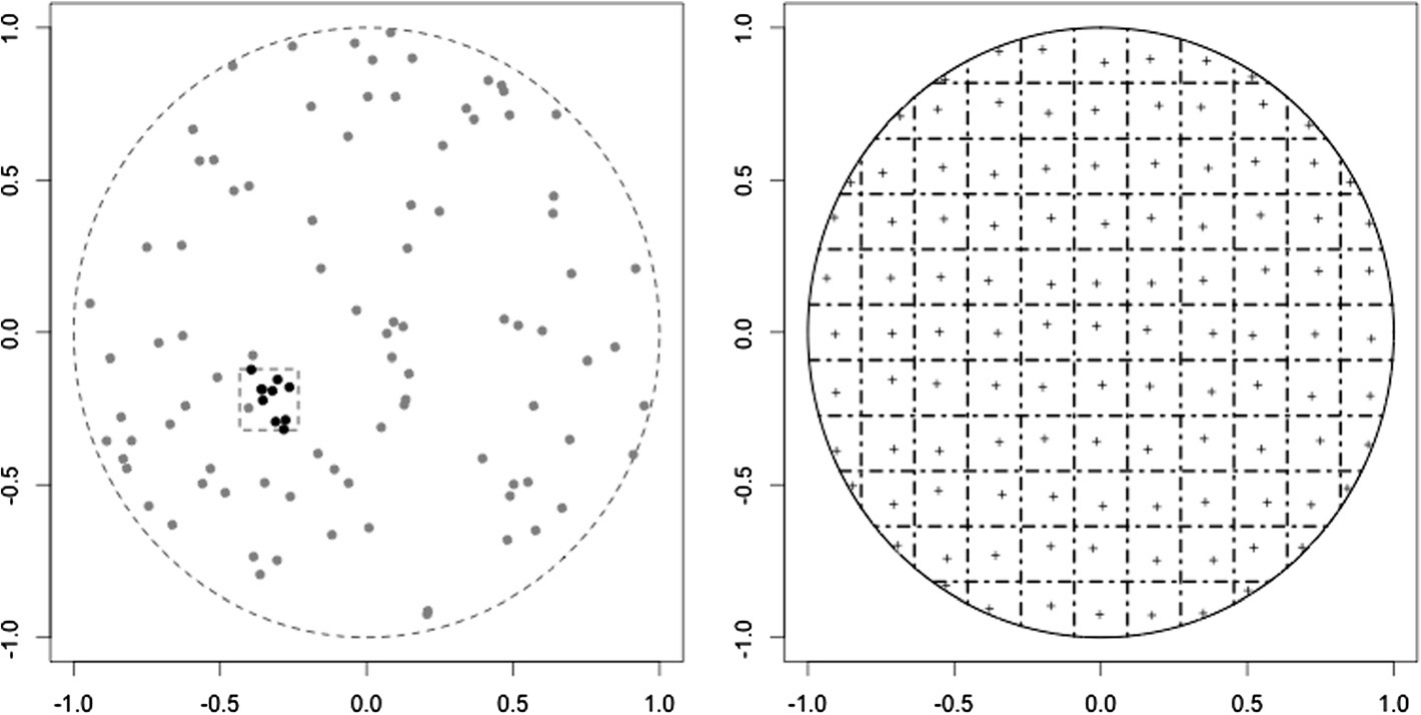
**LEFT: Ninety points (grey) distributed according to a uniform in the unit disk, and 10 additional `outbreak' points (black) from the square left of center.** RIGHT: One level of aggregation for the unit disk, where the exact locations are reassigned to a single point in the corresponding grid square. Each centroid location is represented by a ‘+’.

We consider the uniform distribution on the unit disk as our continuous reference population, with CDF *F*_*0*_ and PDF *f*_*0*_. The unit disk is defined as the two-dimensional surface delimited by a circle of radius 1 centered at the origin. Following our earlier work [7], cases are distributed as a mixture of the reference population and localized increases in the number of cases (i.e. clusters). We write the PDF *f(x)* for cases from a single cluster in subregion *C* of the study region as:

$$f\left(x\right)=0.9*f_{0}\left(x\right)+0.1*g_{0}\left(x\right)$$

where *g*_*0*_ is the uniform distribution on *C*. We choose *C* to be square-shaped, centered at a focus point randomly selected from the region *S*. We fix the geographical extent, which we index by the side length of the square. We denote this index by ‘diameter’ , and consider four values: 0.05, 0.2, 0.35, 0.5. The diameter and mixture of each distribution (here 90%-10%) govern the risk increase in *C*. For example if the diameter is 0.5, the probability of arising in *C* is 2.16 times higher under *f* compared to *f*_*0*_. For each index value, we simulate 1000 datasets of size 100, within which the focus point is the only parameter that varies. The left panel of Figure 1 illustrates one such simulated dataset: 90 cases are drawn uniformly in the unit disk, and 10 are sampled from a small square in the region to represent a cluster.

### Aggregation scheme

Following previous work [7], we create discrete spatial data by aggregating the point data generated with *f*. On the unit disk, we superimpose several regular rectangular grids of varying spacing. For a given grid spacing, spatial locations generated under *f* and *f*_*0*_ are reassigned to the centroid of the corresponding grid square (Figure 1, right panel). Each centroid is slightly off-center to avoid too regular an aggregation and produce a wider range of disease scores. We consider four levels of aggregation, constructed by setting the maximum number of adjacent grid squares to 15, 10, 6, and 4. We index the level of aggregation using the side length of a grid square, with corresponding values 0.13, 0.2, 0.33, and 0.5. Table 1 gives the total number of grid squares where data drawn from *f* can appear.

**Table 1:** Number of grid squares per aggregation level, where side length zero corresponds to continuous data

**Side length**	**0**	**0.13**	**0.2**	**0.33**	**0.5**
*m*	∞	199	88	36	16

### Implementation and evaluation of the mapping

We implement DBM for each side length from Table 1, with *N* = 20 circle points and smoothing parameter *p*_*0*_ = 0.1. We represent the unit disk with *r* = 7827 grid points on the unit disk, and calculate Γ at each point.

To evaluate DBM, we use the metric proposed in Jeffery et al. [17,18]. The mixture *f* dichotomizes risk of disease across the study region, and thus DBM performs well if it locates the high and low risk grid points accurately. Given a threshold γ, the disease score Γ(*y*) > *γ* when *y* is in the cluster area and Γ(*y*) ≤ *γ* when *y* is outside the cluster area. We define two complementary metrics of accuracy: sensitivity, the proportion of grid points in the cluster area that are given a high score, and specificity, the proportion of grid points outside the cluster that are given a low score. We then use a range of values for the threshold *γ* to construct a Receiver Operating Characteristic (ROC) curve and calculate the Area Under the Curve (AUC). The AUC is our final measure of accurate location of the cluster area, ranging from 0 (no cluster area is located) to 1 (the cluster area is perfectly located).

We further investigate performance by exploring where DBM locates a high risk area of ‘reasonable geographic extent’. For each iteration, we first identify the subset of grid points of the study region with a high DBM score (≥ 95th percentile of color palette). Iterations are then classified as successful if: (a) this subset of grid points does not differ from its corresponding convex hull by more than eighty points (~ 1% of all grid points in *S*); (b) the largest distance between any two points of the corresponding convex hull is no more than 1.75 times the side length of a grid square. For each successful iteration, we measure the distances from the center of the located high risk area to the focus point of *C* and to the ‘average centroid’ , and take the difference between these two distances (DiD). For example if the ten points sampled in *C* are distributed among four adjacent grid squares (4, 2, 2 and 4 points respectively), the average centroid would be the average of the four centroids weighted by 0.4, 0.2, 0.2, 0.4 respectively.

## Results

### Application to upstate New York leukaemia data

**Figure 2:**
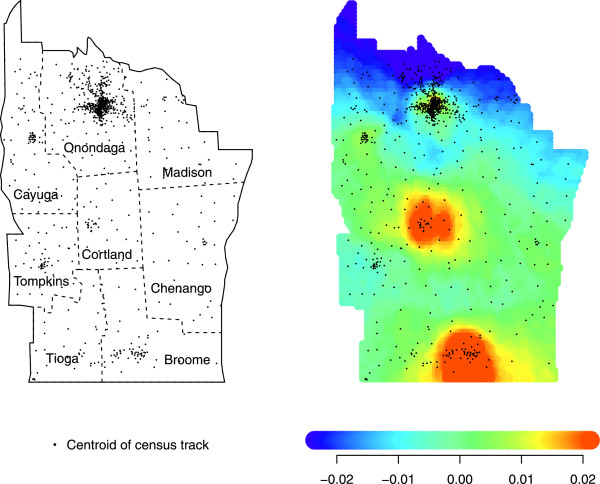
**LEFT: Location of the 790 centroids in upstate New York.** Dotted lines mark county borderlines. RIGHT: Application of DBM (*N* = 40, *p*_*0*_ = 0.1) to the 592 leukaemia cases.

The upstate New York leukaemia data consists of 592 cases found across 790 discrete census block groups. These data are available from StatLib (http://lib.stat.cmu.edu/datasets/csb/) [31]. Each area is represented by the coordinates of its centroid, and the number at risk is given by the 1980 US Census. For 10% of the cases, address was known only within two or three neighboring areas, and thus their contribution was dispersed throughout the concerned areas, weighted according to the census data, which explains some of the non-integer counts [25]. The left panel of Figure 2 shows how centroid locations follow similar variation in population density across the study region. For our application, 592 case locations are used to estimate *f*, while *f*_*0*_ is represented by the census data. The 790 cells correspond to subregions *S*_*i*_, *i* = 1,…,*m* = 790. We choose *N* = 40 circle points and smoothing parameter *p*_*0*_ = 0.1. We superimpose a grid of *r* = 7038 grid points on the study region, and calculate Γ at each grid point. We then use a color scale to map our estimate, shown in the right panel of Figure 2. The color scale is determined by resampling from the reference population *F*_*0*_[17,18]. Low values of *Γ* are in dark blue, moving to green, orange, and then red as the values increase. The resulting mapping shows higher risk in the dense part of Broome county and possibly Cortland county.

### Simulation results

**Figure 3:**
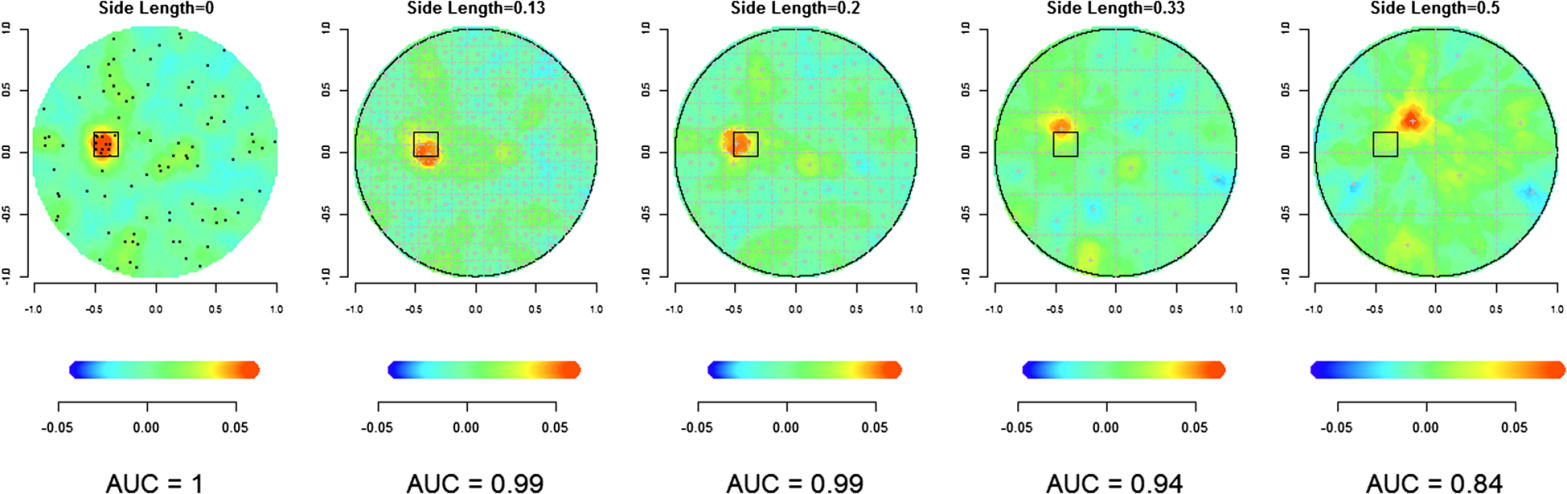
**DBM applied to continuous (Side length = 0) and discrete data (Side length = 0.13, 0.20, 0.33, 0.50) applied with 10 points centered in the dark square (diameter = 0.2) and 90 points uniformly distributed in the unit disk (*N*** **= 20, *p***_***0***_ **= 0.1), and corresponding AUC values.**

We first illustrate mapping for a single simulation using both point data and four levels of aggregation. In Figure 3, we apply DBM to one iteration of our simulations, where the cluster region *C* is defined by the dark square in the left region of the disk (the diameter is set to 0.2). Each of the five panels displays the mapping of these data for one side length value from Table 1, and the value of the AUC is displayed below. Each mapping highlights a region in red, and as the side length increases there is less overlap with *C* while the AUC decreases. In addition, the center of the region highlighted in red is close to the centroid of the grid square that contains the largest portion of cluster area.

**Figure 4:**
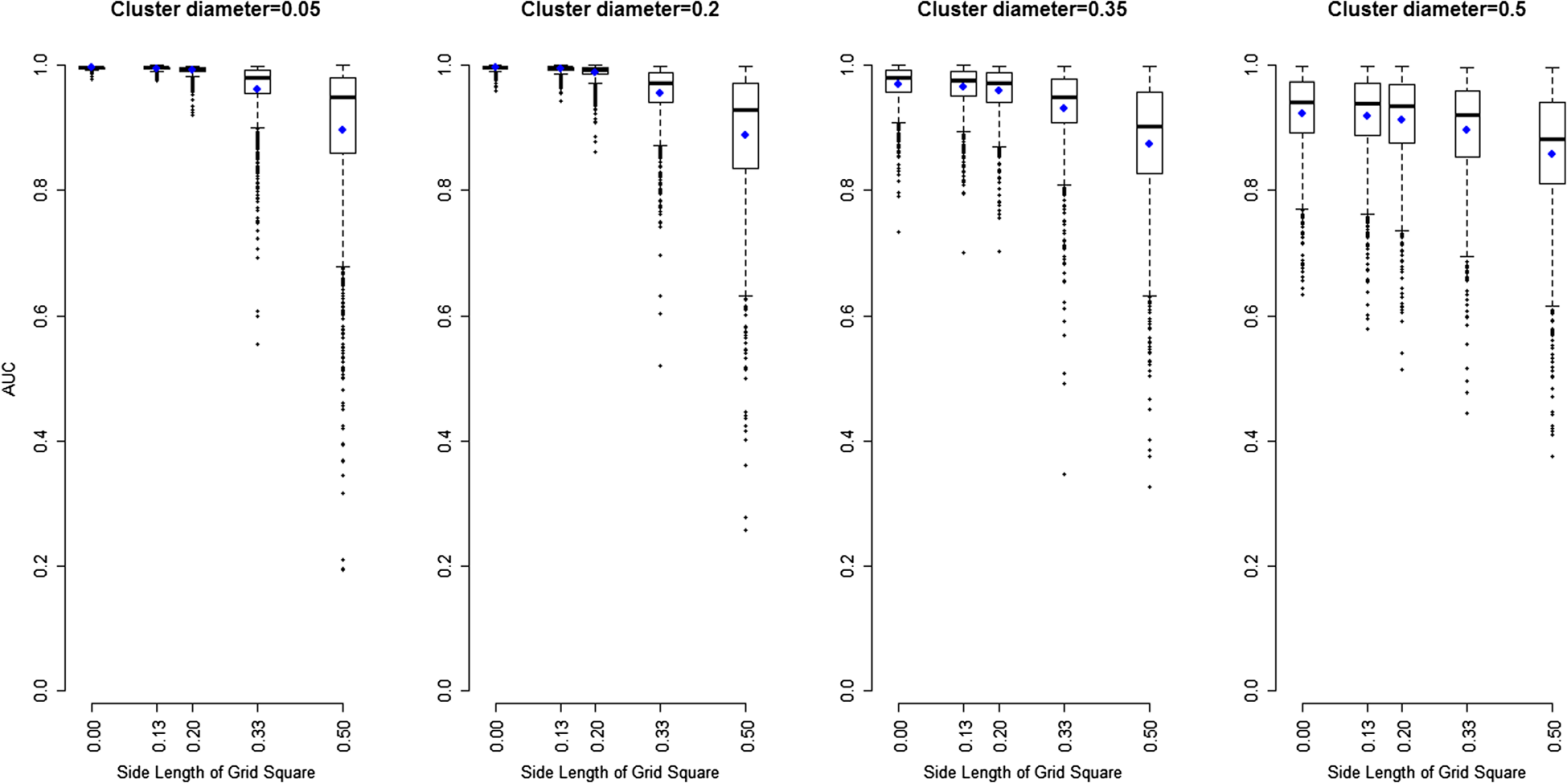
**For each cluster diameter, boxplot of 1000 AUCs (y-axis) to locate high risk area for several aggregation levels (x-axis, side length = 0 for exact location), from 1000 simulations.** Means are represented as blue dots.

Results from 1000 iterations are presented in the four panels of Figure 4. These show the distribution of the 1000 AUCs (y-axis) versus the side length of a grid square (x-axis), for each of the four diameters. A side length equal to zero means the locations are not aggregated (i.e. exact point locations). Each blue dot corresponds to the mean of the 1000 AUCs for one aggregation level. For each diameter, the distribution of AUC remains the same for the lower three side length values. Afterwards, there is a drop in the mean and median, while the interquartile range (IQR) widens much more. For the largest side length, the performance of the mapping appears to be the same regardless of the size of the diameter. Finally, the loss in performance with increasing side length is greater for smaller diameters.

**Figure 5:**
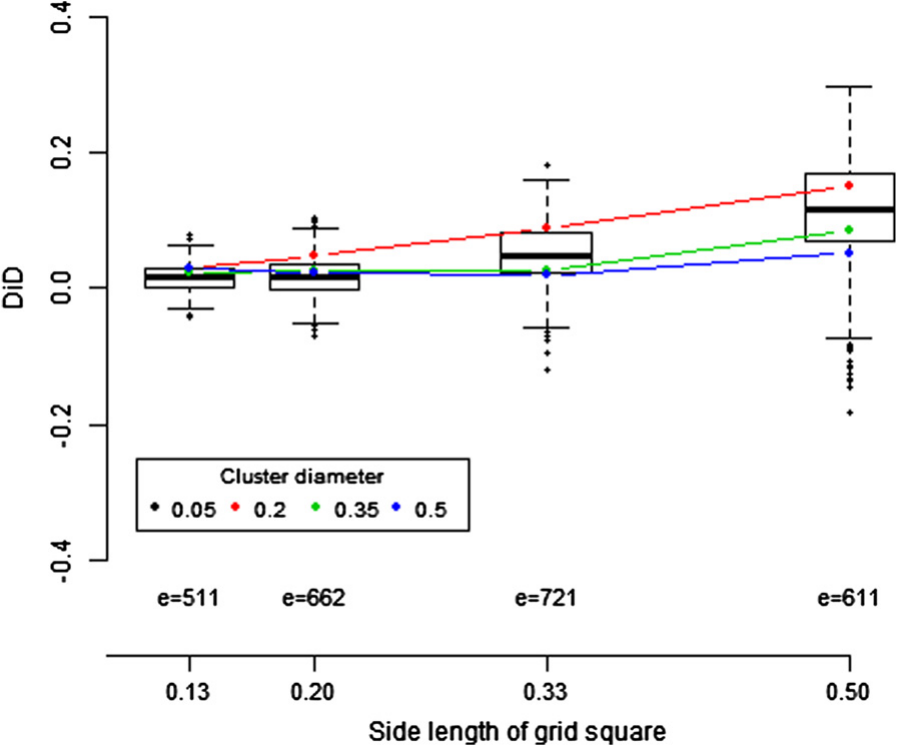
**Distribution of the Difference in Distance (DiD) from the center of the located high risk area to the focus point of the cluster and to the average centroid.** The number *e* is the number of iterations where DBM locates a high risk area of reasonable geographic extent. For cluster diameter 0.2, the distribution is displayed as a boxplot of the *e* DiD values. For each cluster diameter 0.05, 0.20, 0.35 and 0.50, we report the median of the *e* DiD values with a colored dot.

Figure 5 displays the distribution of DiD against the side length of a grid square when the cluster diameter is 0.2. The median DiD values are displayed as a colored dot for each cluster diameters 0.05, 0.20, 0.35, and 0.50. Our results show that at least 50% of the iterations locating a high risk area of ‘reasonable geographic extent’ tend to place it closer to the average centroid than the focus point.

## Discussion

We adapt DBM to a discrete setting, where a centroid point for each aggregation unit represents the location of a case. This allows us to maintain the same approach when mapping either continuous or discrete spatial data. This common framework can lead to study the application of DBM to several datasets simultaneously, when spatial data are available at various levels of resolution [6,32].

We have presented an application of DBM to leukaemia data. Other analyses of this dataset have identified similar hot spots, and raised possible connections with surrounding toxic waste sites [33-35]. We believe that DBM adds a valuable visualization tool to this collection of analyses.

We measure performance of mapping as the correct localization of a cluster placed in the unit disk, using similar metrics to Jones et al. [8]. Simulations show that the accuracy in mapping reduces as resolution of spatial data coarsens, although the decrease in AUC is less dramatic when the high risk area is large. Similar to other studies [7,8,13,36], these results show that the strength of any local change in the spatial distribution of cases will be identified differently depending on the level of discreteness at which the spatial data are available.

The adaptation of DBM to discrete data relies on a choice of centroids and our simulations show that the quality of the mapping is dependent on their location. Our results from Figure 5 suggest that hot spot clusters can be well located when the average centroid is close to the cluster focus point. This occurs when the side length of the grid square is smaller or equal to the cluster diameter, or when the cluster spreads across several aggregated regions [7,8,23]. Waller et al. have also shown that changing the location of the centroid within each aggregation unit can affect the inference (see [23], chapter 4). To accommodate this drawback, some have suggested methods that are *centroid-free*[37-39] including one mapping method where the estimator of the intensity is based on a least square approach weighted by the distribution of the aggregated counts [40]. The effect of centroid choice can be easily studied, and our method could be expanded to be independent of centroid location and instead depend on the support of the aggregation unit by assigning the location of a case randomly in the unit. The random process making such an assignment might be uniform or based on other available data, e.g. topological features. Regardless, the location of case is then represented as continuous and our original DBM method can be implemented. Further study needs to assess how this approach would affect the resulting mapping.

We have limited this study to a uniform distribution in the unit disk and a dichotomized risk. Real data will undoubtedly exhibit complexities of shape and intensity of risk. Yet our simulations allow us to understand the methodology in a simple setting, and focus on the impact of a small number of already important parameters. Concerns about privacy related to electronically collected spatial data are prominently part of the public discourse, which warrants careful and systematic methodological study of the compromises imposed by coarsened data.

## Conclusion

Aggregation of spatial data typically destroys information and thus impedes efforts to monitor these data for spatial disturbances. We have shown that DBM can be implemented for continuous and discrete spatial data. The application to upstate NY data offers new possibilities for its use and visualization of aggregated spatial data. However, the resulting mapping can lose accuracy in the discrete setting and our simulations suggest a complex influence by the interaction between cluster diameter and level of aggregation. The choice of centroids also has an impact on the mapping output. The effect of spatial aggregation on cluster detection, disease mapping, and other useful methods in spatial epidemiology is complex and deserves further study.

## Abbreviations

AUC: Area under the curve; CDF: Cumulative distribution function; DiD: Difference in distances; DBM: Distance-based mapping of disease risk; CoD: Curse of dimensionality; ECDF: Empirical cumulative distribution function; IQR: Interquartile range; MAUP: Modifiable areal unit problem; OR: Odds ratio; PDF: Probability distribution function; ROC: Receiver operating characteristics; RR: Rate ratio; ZIP: Zone improvement plan.

## Competing interests

The authors declare that they have no competing interests.

## Authors’ contributions

All authors conceived of the study and participated in the design. CJ drafted the manuscript and was responsible for statistical programming and data analysis. All authors contributed to the writing of, reviewed and approved the final manuscript.
